# Carbon-based pedicle screw fixation for lumbar fusion for degenerative pathologies in a cancer patient requiring radiotherapy

**DOI:** 10.1093/jscr/rjaf065

**Published:** 2025-02-25

**Authors:** Ralph Jasper Mobbs, Daniel Liu

**Affiliations:** NeuroSpine Surgery Research Group (NSURG), 346 Barker St, Randwick, New South Wales 2031, Australia; Neuro Spine Clinic, Prince of Wales Private Hospital, 346 Barker St, Randwick, New South Wales 2031, Australia; Prince of Wales Private Hospital, 346 Barker St, Randwick, New South Wales 2031, Australia; Faculty of Medicine and Health, University of New South Wales, Randwick, New South Wales 2031, Australia; NeuroSpine Surgery Research Group (NSURG), 346 Barker St, Randwick, New South Wales 2031, Australia; Neuro Spine Clinic, Prince of Wales Private Hospital, 346 Barker St, Randwick, New South Wales 2031, Australia; Prince of Wales Private Hospital, 346 Barker St, Randwick, New South Wales 2031, Australia

**Keywords:** carbon-fibre-reinforced polymer implants, spinal instability, discogenic low back pain, radiolucent pedicle screws, oncological spine surgery, radiotherapy planning

## Abstract

Spinal instability due to degenerative pathologies in patients with metastatic cancer presents significant challenges when spinal fusion and post-operative imaging and radiotherapy are required. Traditional metallic hardware hinders imaging quality and radiotherapy precision due to metal artefact. Carbon-fiber-reinforced polymer (CFRP) implants offer a novel solution, combining mechanical stability with radiolucency to enhance post-operative multidisciplinary care. We present the case of a patient with metastatic cancer requiring spinal stabilization and fusion. To address the dual needs of spinal stabilization and radiotherapy planning, CFRP pedicle screws and rods were used. Postoperative imaging and radiotherapy planning benefited from the radiolucent properties of the implants, enabling precise tumor targeting and disease monitoring. This case highlights the advantages of CFRP implants in managing cancer-associated spinal instability. The radiolucent nature of these implants allows for high-quality imaging and optimal radiotherapy delivery, addressing limitations associated with metallic implants.

## Introduction

Carbon-based pedicle screws, particularly those made from carbon-fiber reinforced polymers (CFRP), have emerged as a significant advancement in spinal surgery, especially for oncology patients requiring spinal stabilization and adjuvant radiotherapy. Spinal instability in patients with cancer, particularly those with metastatic or advanced disease, represents a significant clinical challenge. This instability often necessitates surgical intervention to provide mechanical support, alleviate pain, and preserve neurological function [1]. However, traditional metallic pedicle screws and rods, while effective for stabilization, present challenges in postoperative imaging due to their radiopaque nature, leading to artifacts and scatter in modalities such as computed tomography (CT) and magnetic resonance imaging (MRI) [2]. These imaging distortions can impair the ability to accurately assess tumor margins, detect local recurrences, and assess treatment response [3]. Furthermore, these artifacts can interfere with radiotherapy dose calculations, leading to suboptimal targeting of malignant tissues and potential damage to surrounding healthy structures [4].

The advent of CFRP screws addresses these challenges by offering radiolucency, which substantially reduces imaging artifacts. This property enhances the clarity of postoperative imaging, facilitating precise delineation of anatomical structures and tumor boundaries. For patients undergoing radiotherapy, the improved imaging quality allows for more accurate targeting of radiation beams, thereby sparing adjacent healthy tissues and potentially reducing radiation-induced complications [3]. Biomechanically, CFRP screws provide comparable strength and stability to their metallic counterparts, ensuring effective spinal fixation without compromising structural integrity [5, 6]. Their compatibility with advanced imaging techniques and therapeutic interventions makes them particularly advantageous in the multidisciplinary management of spinal tumors, where both surgical stabilization and precise radiotherapeutic targeting are critical [3].

In summary, the integration of carbon-based pedicle screws in spinal oncology offers dual benefits: maintaining mechanical stability and enhancing the efficacy of imaging and radiotherapy. This advancement represents a significant step forward in the comprehensive treatment of spinal malignancies, aligning surgical objectives with oncological needs to improve patient outcomes.

## Case report

**Figure 1:**
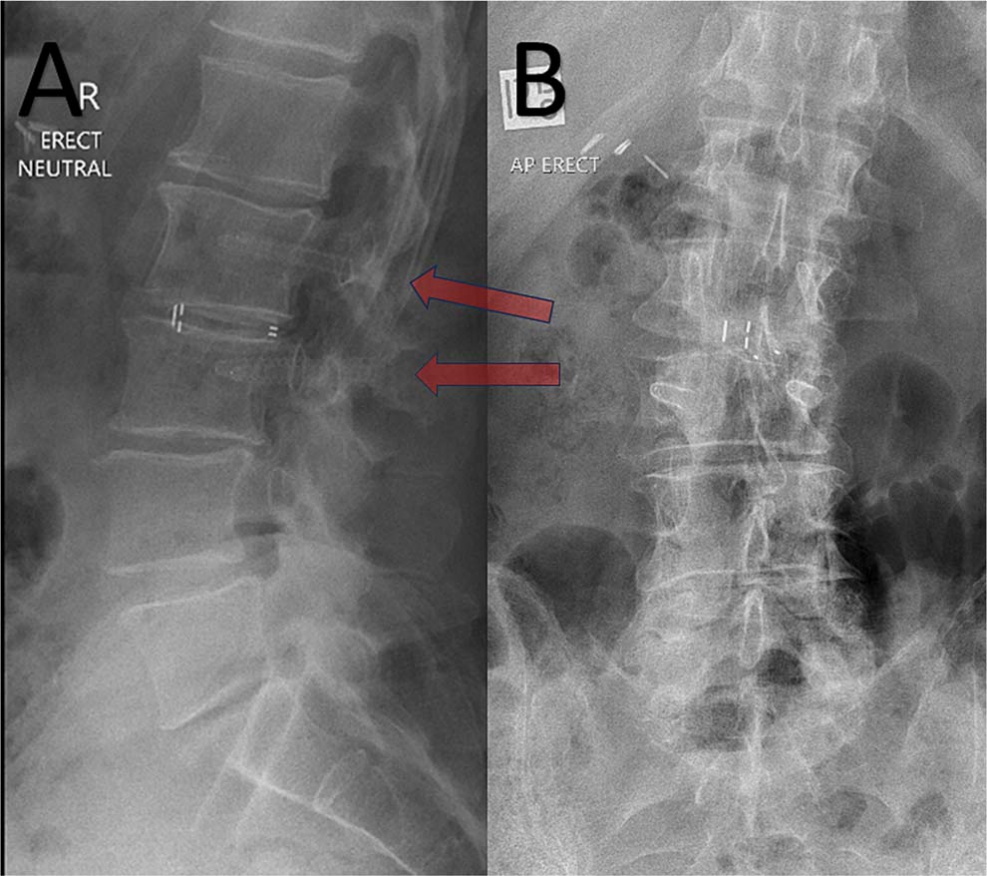
Post-operative erect XR lumbar spine after L2/3 lumbar fusion. Arrow = CFRP pedicle screws. A – Sagittal; B – Anteroposterior views.

A 72-year-old male with a history of bowel cancer presented with severe discogenic back pain and lumbar instability at L2/3 requiring surgical intervention. The patient had previously been diagnosed with bowel cancer and although was clear of tumor load after prior surgery, the oncology team informed that further recurrence of disease was likely, and potentially a candidate for adjuvant radiotherapy and chemotherapy in the future. Given the potential need for repeat imaging and precision radiotherapy, the oncology and surgical teams opted for spinal fixation with carbon-based pedicle screws and rods. The patient underwent an uneventful L2/3 posterior lumbar fusion with the placement of carbon-fiber pedicle screws (Matrix Medical Innovations, Australia) and PEEK interbody implant (ASpine, Taiwan). Postoperative imaging, including the attached X-ray (Fig. 1), and demonstrated excellent screw positioning and spinal alignment. The radiolucent nature of the screws resulted in minimal disruption of the radiographic field, a stark contrast to the typical imaging artifacts associated with metallic implants. The patient experienced an uneventful recovery, with resolution of his discogenic back pain and no perioperative complications. Follow-up imaging showed continued alignment and no radiographic interference, allowing seamless planning for subsequent radiotherapy should this be necessary.

The patient has provided informed consent for his data to be included in this study.

## Discussion

Spinal instability and pain in patients with potential cancer to the spine, or surrounding structures, represents a unique challenge in balancing mechanical stabilization with effective ongoing cancer treatments. The radiopaque nature of traditional metallic hardware interferes with the accurate interpretation of imaging modalities including CT and MRI due to its metal artefact [2]. This can result in impaired precision in radiotherapy planning and tumor surveillance [3]. Additionally, metallic implants can cause scattering and artefacts during radiotherapy, potentially compromising treatment accuracy [4], and dose delivery.

Carbon-based spinal implants are a promising solution for patients requiring multimodal cancer treatment. Their radiolucent properties preserve imaging clarity, which is critical for accurate radiotherapy delivery and longitudinal oncological monitoring [7]. Additionally, their biomechanical properties are comparable to traditional metallic devices, ensuring stable spinal fixation without compromising surgical outcomes [5].

In this case, the use of carbon-based screws facilitated both effective spinal stabilization and enhanced radiotherapy planning should this be necessary, providing a dual benefit. The postoperative imaging (Fig. 1) underscores the radiological advantages of these implants, as the screws are nearly invisible, preserving the clarity of adjacent structures.

The use of CFRP pedicle screws in degenerative pathologies remains a relatively novel technique, with recent literature demonstrating promising results. CFRP rods have been shown to have mechanical properties, including mean bending yield load, bending ultimate load, and stiffness, that are comparable to currently utilized titanium-based systems [8]. In human osteoporotic spinal cadaver models, CFRP demonstrated similar microscopic loosening compared to its titanium counterparts [9]. Additionally, there have been several retrospective series describing the safety and noninferiority of CFRP compared to titanium hardware. These studies have demonstrated that there were no significant differences between the two groups in terms of fusion rates or post-operative hardware-related complications including loosening, fracture, or other failure [10–12]. CFRP hardware has also demonstrated excellent outcomes in degenerative spinal disease requiring spinal fusion, offering similar advantages compared to titanium alternatives [13, 14]. Although these studies have demonstrated favorable outcomes with CFRP implants in oncology patients, large-scale studies are still lacking. Therefore, the understanding of the long-term mechanical durability of CFRP hardware in diverse patient populations remains limited. This case report adds to the growing body of literature by presenting a case of a successful integration of carbon-based fixation in a multidisciplinary oncological treatment plan.

Additionally, the use of radiolucent implants has implications for cost-effectiveness in healthcare. Although CFRP implants have a higher initial cost, by reducing the need for repeat imaging or adjustment in radiotherapy planning, these implants may streamline care pathways and reduce overall healthcare expenditures, particularly in high-risk cancer populations [15].

## Conclusion

This case highlights the benefits of carbon-based pedicle screws in patients requiring spinal stabilization alongside cancer treatments such as radiotherapy. The radiolucent nature of these devices enables precise imaging and treatment planning while maintaining robust mechanical stability. As cancer survival rates improve, the integration of advanced materials like carbon-fiber implants into oncological care is poised to enhance multidisciplinary treatment outcomes.
